# Multi-Shaded Edible Films Based on Gelatin and Starch for the Packaging Applications

**DOI:** 10.3390/polym14225020

**Published:** 2022-11-19

**Authors:** Iftikhar Ahmed Channa, Jaweria Ashfaq, Muhammad Ali Siddiqui, Ali Dad Chandio, Muhammad Ali Shar, Abdulaziz Alhazaa

**Affiliations:** 1 Thin Film Lab as Part of Materials and Surface Engineering Group, Department of Metallurgical Engineering, NED University of Engineering and Technology, Karachi 75270, Pakistan; 2 Department of Mechanical & Energy Systems Engineering, Faculty of Engineering and Informatics, University of Bradford, Bradford BD7 1DP, UK; 3 Department of Physics and Astronomy, College of Science, King Saud University, Riyadh 11451, Saudi Arabia

**Keywords:** edible film, biodegradable film, WVTR, surface morphology, hardness

## Abstract

Starch and gelatin are natural biopolymers that offer a variety of benefits and are available at relatively low costs. In addition to this, they are an appealing substitute for synthetic polymers for the manufacturing of packaging films. Such packaging films are not only biodegradable but are also edible. Moreover, they are environmentally friendly and remain extremely cost-effective. In lieu of this, films made from fish gelatin and cornstarch have been the subject of several experiments. The pristine gelatin films have poor performance against water diffusion but exhibit excellent flexibility. The goal of this study was to assess the performance of pristine gelatin films along with the addition of food plasticizers. For this purpose, solutions of gelatin/cornstarch were prepared and specified quantities of food colors/plasticizers were added to develop different shades. The films were produced by using a blade coating method and were characterized by means of their shaded colors, water vapor transmission rate (WVTR), compositional changes via Fourier transform infrared spectroscopy (FTIR), hardness, bendability, transparency, wettability, surface roughness, and thermal stability. It was observed that the addition of several food colors enhanced the moisture blocking effect, as a 10% reduction in WVTR was observed in the shaded films as compared to pristine films. The yellow-shaded films exhibited the lowest WVTR, i.e., around 73 g/m^2^·day when tested at 23 °C/65%RH. It was also observed that the films’ WVTR, moisture content, and thickness were altered when different colors were added into them, although the chemical structure remained unchanged. The mechanical properties of the shaded films were improved by a factor of two after the addition of colored plasticizers. Optical examination and AFM demonstrated that the generated films had no fractures and were homogeneous, clear, and shiny. Finally, a biscuit was packaged in the developed films and was monitored via shore hardness. It was observed that the edible packed sample’s hardness remained constant even after 5 days. This clearly suggested that the developed films have the potential to be used for packaging in various industries.

## 1. Introduction

Foods’ resistance to changes in temperature, time, and physical forces is impacted by the fact that they are composed of living tissues [1,2]. Physical, chemical, and biological factors can cause food to deteriorate [3,4]. Food is meant to be packaged to prevent it from physical, contaminant, and microbial harm. Foods are packed, and a distinct atmosphere is formed surrounding them, to reduce food spoiling and increase the lifespan [3,5,6]. The usage of plastic as a packaging material has increased globally in recent decades, yet because it cannot biodegrade, it can have major negative effects on the environment [7,8,9]. A possible option that represents a cheap, edible alternative with a good degradation rate and qualities similar to ordinary plastics is the creation of films made from biodegradable materials. This is because using environmentally friendly materials can help with solid waste collection issues [10].

The three benefits of using edible films and coatings are their ability to separate the food from its environment, to stop food waste and degradation [11,12], and to increase the food’s storability [13]. Consumable food product storage and transportation are crucial. The food must be delivered to the customer during these procedures with the least amount of quality loss possible [14]. During transportation, numerous factors can cause food deterioration, such as oxidation [15], moisture diffusion [16], UV-derived food degradation [17,18], dust accumulation, and hygiene-related issues [19]. Hence, a quality packaging can play a key role in retaining the food quality [1,20]. Layers prepared out of different substances and placed on the product in the form of a thin film with the intention to be consumed with the product are known as edible films and coatings [21]. One of the newest methods for ensuring food safety is edible film technology, which was created as an alternative to common packaging materials including glass, tin, and polymers for active packaging [22,23]. The U.S. Food and Drug Administration has given edible films and coatings the GRAS (generally recognized as safe) designation [24]. Many components may be used to create edible films and coatings. In general, a film-forming substance such as protein (gelatin, protein, soy protein), polysaccharides (starch, chitosan), or lipid could be employed, and solvents such as water, ethanol, and acetone can be used. Researcher may use plasticizers, surfactants, and additives in a film or coating solution, including essential oils, antioxidants, antimicrobials, flavoring agents, colorants, vitamins, and chemical preservatives [25].

Because of its low cost, abundance, functional properties (flavorless, tasteless, odorless), optical (transparent, colorless), and barrier qualities (carbon dioxide and oxygen permeability), starch biopolymer has received a lot of attention due to its film-forming abilities [26]. These characteristics are suitable for film generation; however, starch biopolymers have poor mechanical and water vapor permeability (WVP) qualities. Researchers have recently been examining the integration of biopolymers and other additives utilized during film creation to improve the WVP and mechanical characteristics of starch biopolymer-based films [7,27]. Therefore, by using biopolymers or several other substances that are water insoluble and/or have antibacterial qualities, including essential oils and glycerol, starch films’ physical and mechanical attributes can be enhanced. A straightforward, flavorless, odorless, viscous polyol molecule called glycerol is cooked at a high temperature and then freezes to create pasta [28,29]. According to its stability with starch, which encourages high hardness characteristics by interfering with starch packing and reducing the intermolecular interactions between the starch molecules, glycerol is frequently employed as a plasticizer for starch films [2,30,31].

Gelatin can be used to improve the barrier qualities of the edible film. Gelatin is a partially degraded byproduct of collagen found in animal connective tissue. It is a white or yellowish, transparent, glossy solid. Gelatin’s functional properties, which include its capacity to bind water, create gels, operate as a water vapor barrier, produce films and foams, and tend to emulsify, make it useful in the food, pharmaceutical, photographic, and cosmetics sectors [32,33]. Strong gas barrier qualities and swelling behavior are characteristic of gelatin. A.A. Al-Hassan and M.H. Norziah [34] demonstrated that gelatin, glycerol, and cornstarch are excellent examples of materials with strong mechanical and physical qualities. Tensile strength ranges from 1.28 MPa to 18.06 MPa. There are many additives used to enhance color, flavor, or appearance and to extend shelf life. The food colorant serves as an additive and may enhance the stability. Not only this but creating multi-shaded packaging films is in itself pleasing to eye. Therefore, chemicals called food colorants are added to food matrices in order to improve or preserve the looks of foods, which can be altered or lost during processing or storage. Because they may offer additional health advantages, natural colorants are currently replacing synthetic colorants [35]. In a laboratory setting, the blade casting process is the most commonly used approach for producing edible materials since it is both inexpensive and simple.

The aim of the study was to develop environmentally friendly edible and biodegradable packaging systems based on gelatin–cornstarch (G+CS) blending combined with various food colors, such as red (G+CS+R), green (G+CS+G), blue (G+CS+B), and yellow (G+CS+Y) using glycerol as the plasticizer and to evaluate their chemical, mechanical, surface, barrier, and optical properties. Food coloring was added along with cornstarch, gelatin, and other ingredients in order to improve the product’s look and properties. This is the first study to demonstrate the use of food coloring in conjunction with gelatin and cornstarch to create edible films.

## 2. Method and Materials

### 2.1. Materials

The edible film consisted of starch from corn (CAS # 9005-25-8 and purchased from Sigma Aldrich, St. Louis, MO, USA), gelatin from cold water fish skin (CAS # 9000-70-8 and purchased from Sigma Aldrich, St. Louis, MO, USA), glycerol (CAS # 56-81-5 with 99.0% purity and purchased from Sigma Aldrich, St. Louis, MO, USA), and food color purchased from Vibgyor Company, Karachi, Pakistan.

### 2.2. Preparation of Solution

**Figure 1:**
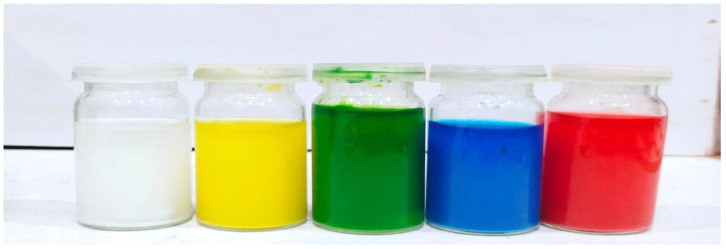
Visual appearance of the film-forming solutions in white, yellow, green, blue, and red color.

The starch solutions were made by dissolving 5 g of maize starch in 100 mL of distilled water for 45 min. After obtaining a fully dispersed white solution, 10 g of gelatin was dissolved in a cornstarch solution for 1 h at room temperature. Following this interval, the solution was heated to 70 °C and held at this temperature for 10 min to produce a clear solution. The plasticizer (glycerol) was then added, with constant gentle stirring for 10 to 20 min to prevent air bubbles and gelatin denaturation. Similarly, 5 solutions were created to add 5 distinct food colors. The food color was added to the prepared solutions and stirred at a constant speed for 20 min to ensure homogenous mixing. The solutions were prepared and are shown in Figure 1.

### 2.3. Preparation of Edible Film

A doctor blade (ZAA 2300, Zehntner Testing Instruments, Sissach, Switzerland) was used to generate edible films on clear PET substrates (Melinex^®^ ST504, DuPont Teijin Films UK Ltd., Middlesbrough, UK). At 40 °C, the solution was dried for 24 h. The films were pulled off to create free-standing films (Table 1). These films were preserved in a vacuum desiccator until further characterization of the specimens.

### 2.4. Characterization

#### 2.4.1. FT-IR Analysis

An FT-IR machine (Bruker ALPHA-P, Karlsruhe, Germany) with a wavelength range from 500 cm^−1^ to 4000 cm^−1^ was used to examine the edible films. At 4 cm^−1^ resolutions, 64 scan summations were used to obtain the spectra.

#### 2.4.2. UV-VIS

A UV-VIS spectrophotometer (Shimadzu UV-1800, Shimadzu Deutschland GmbH, Duisburg, Germany) operating between 200 nm and 800 nm was used to measure the transparency. 

#### 2.4.3. Contact Angle

Water droplets were used to determine the contact angle using a contact angle goniometer (SL200A) made by KINO Scientific Instrument Inc., Boston, MA, USA.

#### 2.4.4. Bending and Hardness

The edible film was bent using a custom-built cyclic curve analyzer, cycling the film in a customized twisting range with one end fixed and the opposite end moving to and fro. The sample dimensions for this were about 6 cm × 10 cm. For WVTR test, a sample was cut from the middle of the bent film. The test was carried out on at least three samples for the statistical analysis. An Anton Paar nanoindentation hardness tester with a diamond indenter was used to measure the hardness of the edible films. A maximum load of 10.0 mN was applied at a rate of 20 mN/min. The Poisson ratio was 0.50 throughout the whole film. At least five indentations were made for each film as per the ISO 14577 guidelines for performing nanoindentations.

#### 2.4.5. Water Vapor Transmission Rate (WVTR)

A standard aluminum cup that met with ASTM standard E-96 was donated by Thwing-Albert Instrument Company (West Berlin, NJ, USA). The test was conducted using the technique described by Channa et al. [36]. For each variation, at least three samples were tested in the same prescribed conditions for a better statistical analysis.

#### 2.4.6. Thermogravimetric Analysis (TGA)

TGA investigated the thermal stability of the films using a thermogravimetric analyzer (SDT Q-600 TA Instruments, Artisan Technology Group, Champaign, IL, USA). The initial sample weight for each procedure was set at 5–8 mg. The samples were heated from 24 to 550 °C at a rate of 10 °C/min in a nitrogen atmosphere using a low flow rate of 200 mL/min.

#### 2.4.7. Atomic Force Microscopy (AFM)

Atomic force microscopy was used to analyze the films’ surface morphology. The fundamental idea behind AFM is to measure the forces acting on a cantilever and the sample surface (in the x-y plane), which is then converted into a cantilever deflection according to Hooke’s law and is shown as a topographic picture with a fixed scan size (10 mm × 10 mm). The dynamic SPM acquisition mode and contact SPM acquisition mode were used to evaluate the obtained pictures (AFM, ZLAFM. Micro-Nano Equipment Co., Ltd., Shanghai, China). Finally, Rq, Ra, and Z, and the formula provided in the paper by Zuo. G et al. [37], were used to determine the roughness of the film surface.

#### 2.4.8. Shore Hardness

Shore hardness determines a material’s hardness. An analogue TECLOCK GS-702G Shore Durometer was used for that experiment. For the real-time testing, the packaged samples were stored in certain conditions (25 °C and 40%RH) and the shore hardness was analyzed.

## 3. Results and Discussion

### 3.1. Composition Analysis

**Figure 2:**
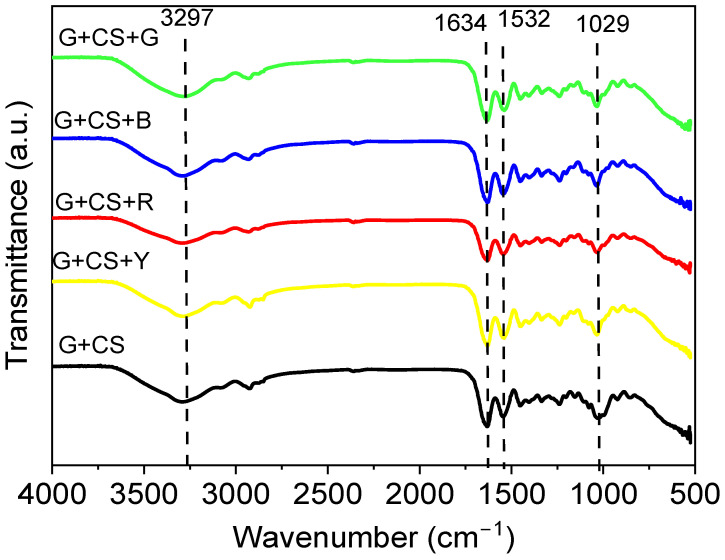
Fourier transform infrared (FTIR) spectrum of films. The black line denotes G+CS, the yellow line denotes G+CS+Y, the red line denotes G+CS+R, the blue line denotes G+CS+B, and in the green line denotes G+CS+G.

The functional groups in gelatin, cornstarch, and food color edible films were identified via an FT-IR analysis of the bands produced by the spectra. The absorption spectra of the edible films made from gelatin and cornstarch are shown in Figure 2. Both the stretching vibration of the O-H groups and the intermolecular hydrogen bonding of gelatin are represented in the wide spectrum range at 3281 cm^−1^. For either amide II or primary amine, the absorption peak at 2877 cm^−1^ corresponds to the stretching vibration of the C-H bond. In the edible films made of gelatin and cornstarch, the C-O groups stretching is represented by the peak with the highest intensity, i.e., 1021 cm^−1^. At 1408 cm^−1^ and 1600 cm^−1^, two distinctive absorption bands were found, demonstrating the symmetric and asymmetric stretching vibration of COO- group, respectively. Contrarily, the FT-IR spectra of the modified film did not alter as a result of the addition of food color to the cornstarch- and gelatin-based film. In addition, a path could be created for water molecules to pass through [38]. The addition of glycerol and a reduction in hydrogen interactions help to create an elastic edible film and to reduce its stiffness. The food color material added from the direct mixing process does not produce a new functional group. It induces the bioplastic material to become water soluble even at this stage. In the bioplastic materials that underwent FT-IR testing, there were also carbonyl (CO) and ester (COOH) functional groups, which allow these bioplastic materials to degrade [39]. Furthermore, the FT-IR results are in perfect agreement with the work done by Wang et al. (2017) [40].

### 3.2. Optical Analysis

Protection from UV radiation has a significant impact on food quality. Food sources that have undergone oxidation may have changed flavor, lost nutritional content, or developed hazardous chemicals, all of which make them less appealing to consumers or inappropriate for their needs. Lipid oxidation is accelerated by the oxygen created by the sun’s UV light [41]. Furthermore, high energy UV rays might promote polymeric chain deterioration within the food. This could also cause oxygen diffusion through deteriorated chains [42]. The oxidation rate under the light is significantly enhanced even at low temperatures as deteriorated spots may serve as diffusion points for oxygen, and as a consequence, processed packaged food suffers and degrades [43]. To stop UV rays from oxidizing food, packaging films should have UV screening capabilities [18,44]. In order to prevent food quality degradation brought about by physical compound changes or synthetic reactions, biopolymer coatings have been used. Gelatin has recently been used due to its excellent barrier qualities [45]. Its stability and other qualities are increased by the addition of glycerol and food coloring.

**Figure 3:**
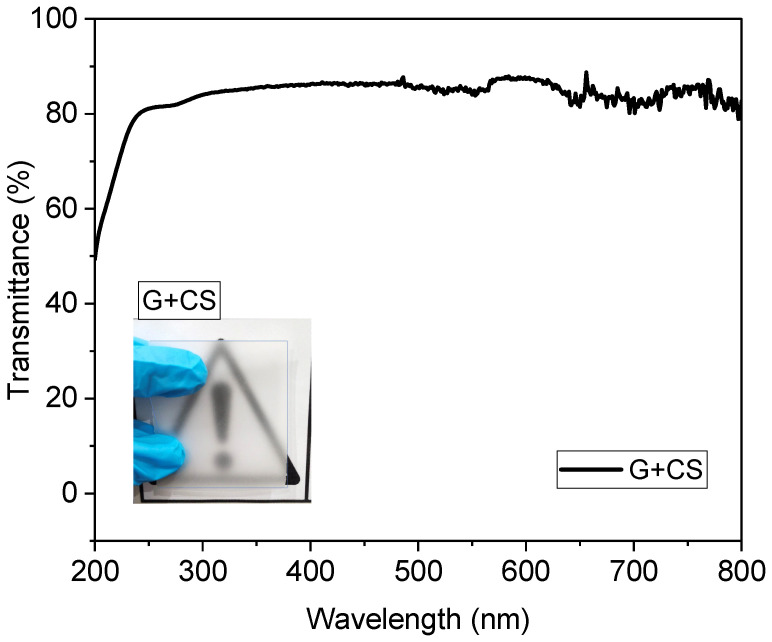
Transmittance spectra of gelatin–cornstarch-based edible films (the black line indicates G+CS film) and the inset shows a pictorial view of the same films.

**Figure 4:**
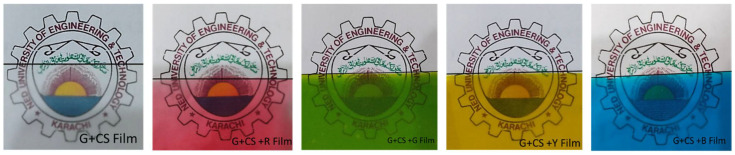
Transparency of the edible films (the black line indicates the film area). The background image is the logo of NED University of Engineering and Technology.

Figure 3 and Figure 4 are concerned about the transparency of the films wherein effect of addition of food color to starch–gelatin films is shown. The transparency of food color gelatin–starch films was remarkably similar to that of gelatin–starch films. Additionally, the colorant in the film affected the film’s opacity. Without food colorants, the film provided the strongest barrier to light transmission at wavelengths of 600 nm (as shown in Figure 3). However, since food coloring does not completely absorb light, its presence reduces the transparency of the film. This may have been the result of the gelatin–starch-based film’s structural alterations and the presence of a crystalline material on its surface. It was initially proposed that the amount of crystals in a sample and the compressed structures of the polymer chains, which impeded light transmission, had a significant impact on the opacity [46].

### 3.3. Wettability Properties

**Figure 5:**
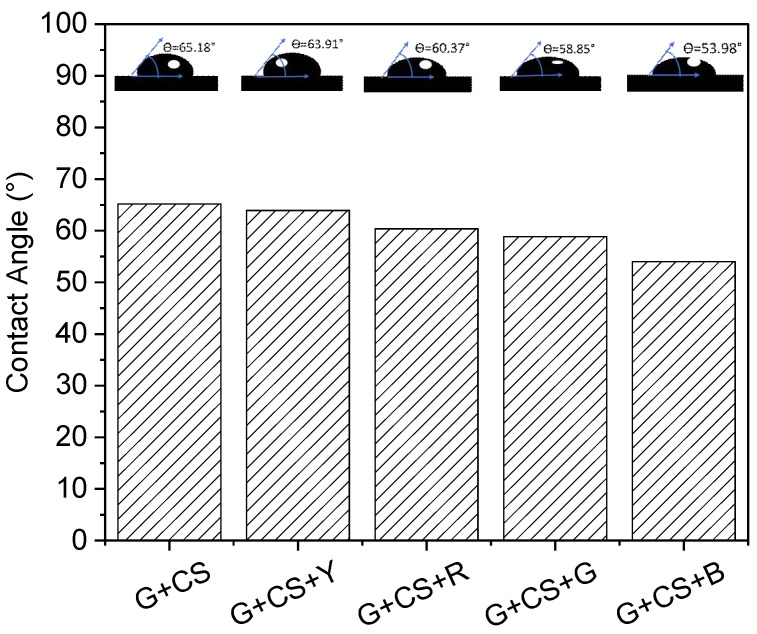
CA (contact angle) image of different concentrations of food color in gelatin–cornstarch-based edible films.

The contact angle revealed whether a surface was hydrophilic or hydrophobic. The performance of the gelatin–starch-based film was characterized by surface wettability, moisture content, and water vapor permeability. The static water contact angle readings provided useful information regarding the solid’s wettability and how well it was wetted by water. Figure 5 displays the values of the water contact angle (CA) and the calculated reading. For example, the CA readings represented the surface’s hydrophilicity (CA 90°) or hydrophobicity (CA > 90°). [10]. Since the fundamental disadvantage of a gelatin–starch-based film is its affinity to water, which restricts its application, a more hydrophobic surface is preferred. Figure 5 shows the pictures of the water CA on the gelatin–starch-based films. The photographs clearly illustrate that the gelatin–starch-based film with food coloring has a lower hydrophilicity and the plain gelatin–starch-based film has a higher hydrophilicity. The findings indicate that, for the gelatin–starch-based films, the water CA values varied from 70° to 50°. For the gelatin–starch yellow color film, we hypothesize that variations in food colorant levels, starch molecule size, and polymerization levels were contributing factors to variations in the water contact angle [20,47].

### 3.4. Flexibility of Film

**Figure 6:**
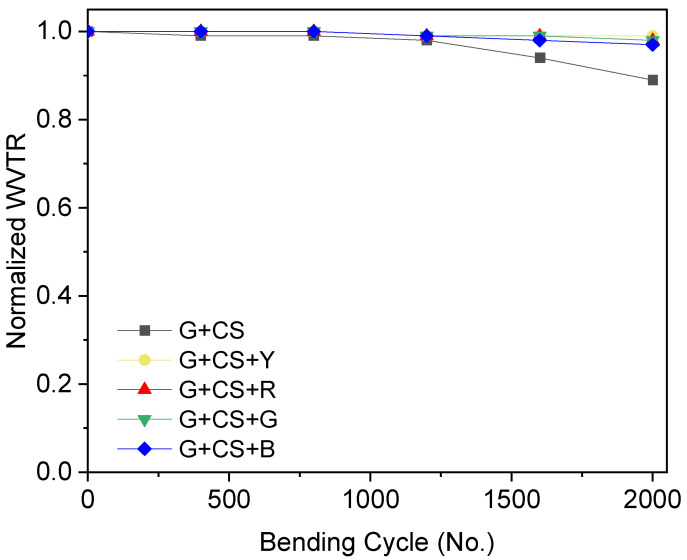
Normalized WVTR value of gelatin–cornstarch-based edible films with red, blue, green, and yellow food color plotted against the number of bending cycles at a bending radius of 6 cm. The black curve represents the pristine G+CS film, the red curve represents the G+CS+R film, the blue curve represents the G+CS+B film, the yellow curve represents the G+CS+Y film, and the green curve represent the G+CS+G film vs. the number of bending cycles. All the rested films had a thickness of 100 µm.

To encapsulate different foods, barrier materials must also be bendable without causing damage. Films made of gelatin, cornstarch, and food color should not be brittle or hard because of this. In order to maximize the food colorant and its effect on flexibility, a bending test was carried out on films with various food colorings. Figure 6 displays the bending performance. A 6 cm bending radius was used during the bending test. Figure 6 makes it quite evident that adding more food colors increases flexibility while ensuring the original WVTR of bent films does not deteriorate. A comparison of the films’ bending ability is shown in Figure 6 [48]. The pure gelatin–cornstarch films lost their original WVTR values after 10,000 bending cycles. The gelatin–cornstarch film’s ability to operate as a water barrier was diminished by 5–25% of their starting value even after 10,000 bending cycles. This is because the adhesive bonding produced by the protein substance was slightly reduced. The film made from gelatin-based cornstarch and food coloring maintained its flexibility and did not exhibit any negative bending-related impacts on WVTR. The flexibility of the film increased as compared to the clean film due to the plasticizing effects of food coloring. As a result, the food color, gelatin, and cornstarch exhibited a strong adherence to one another, and the drop in WVTR was not very considerable. The food color and gelatin have a strong bond, therefore, when the films were bent, they became twisted, but when the pressures were released, they reverted to their original orientation. While bending enabled moisture to drop and increased the overall permeability of the film, it may also have produced a minor fall in WVTR [49].

### 3.5. Analysis of Nanoindentation

**Figure 7:**
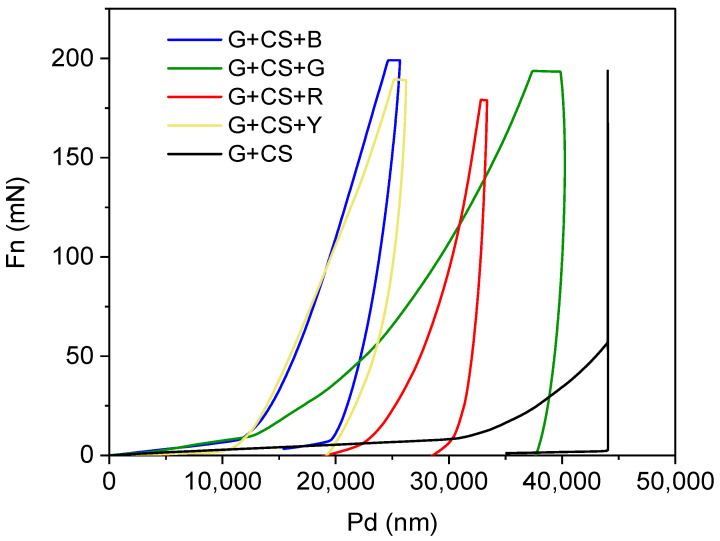
Nanoindentation curve of edible films plotted against force vs. displacement. The G+CS+R film is indicated by the red line, the G+CS+B film is indicated by the blue line, the G+CS+G film is indicated by the green line, the G+CS+Y film is indicated by the yellow line, and the simple G+CS film is indicated by black line.

Figure 7 displays the pure gelatin–cornstarch film’s load penetration depth curves and how they vary with red, blue, yellow, and green food colors. The measurements demonstrate that the pristine gelatin–cornstarch film is moderately hard and has a maximum depth of penetration of 150 Mn and above. This explains the food colorant films’ softness in a very simple way. The penetration depth of the gelatin–cornstarch-based films slightly decreased and occurred in the range from 10k to 40k when food colorant was incorporated. Food coloring was added, but it did not make the films harder; as a result, the films exhibited resistance to penetration. For all of the samples with various loadings of food colors, a very similar response was seen. Higher plasticizer and starch concentrations in the gelatin film might be the cause of this finding. This indicates that adding food color to a gelatin-based film made of cornstarch and gelatin results in films that are neither as soft nor as hard as the G+CS film (the comparable indentation is shown in Figure 7) [50,51].

### 3.6. Surface Analysis

**Figure 8:**
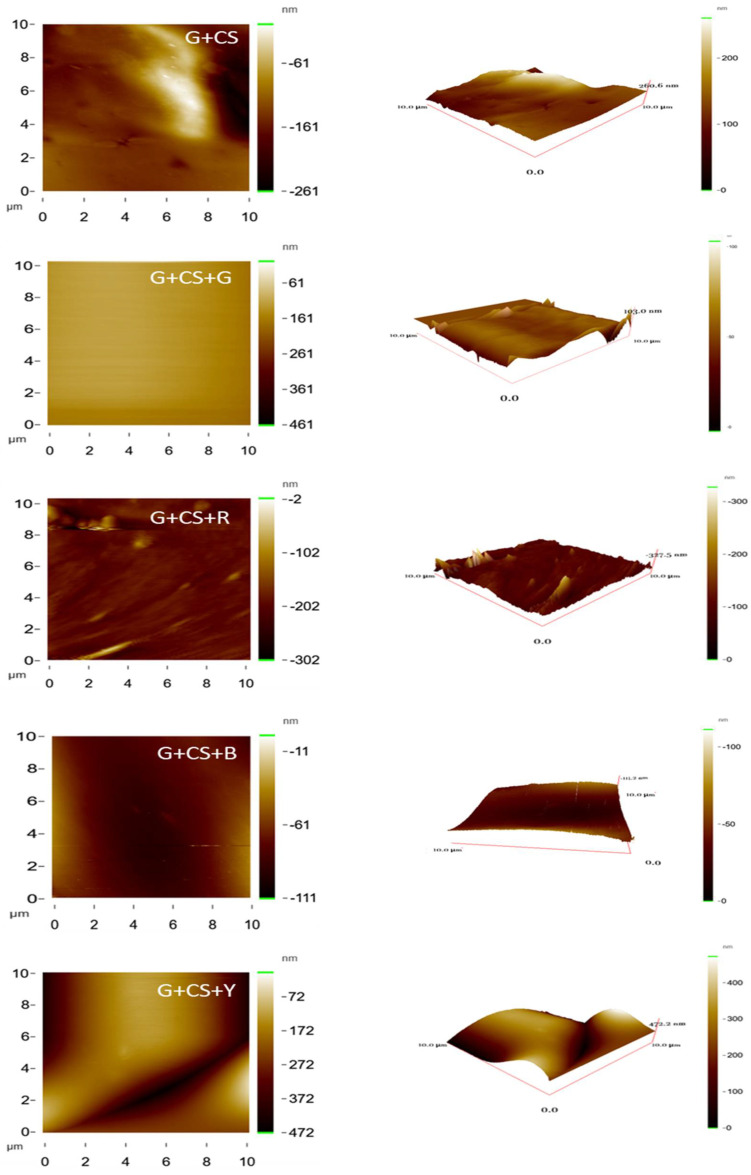
Typical AFM images showing the two-dimensional structure of AFM images of gelatin–cornstarch-based edible films with and without food color and showing the three-dimensional structure of AFM images of gelatin–cornstarch-based edible films with and without food color.

In order to better examine the surface morphology of various films, the atomic force microscopy (AFM) technique is an effective tool for providing qualitative and quantitative information on the nanoscale. In order to illustrate the morphology (a qualitative parameter) and roughness (a quantitative parameter) of several edible films, bi- and tri-dimensional AFM pictures are shown in Figure 8. The highest bulges were found in the yellow color gelatin–cornstarch films (G+CS+Y) with food color. Red, blue, and green food coloring added to edible films resulted in a reasonably smooth and continuous matrix with lower porosity and fewer defects (Figure 8). However, when several foods colors were added, the films’ surface roughness increased (Figure 8). Consequently, the smoothness of the film surface was enhanced without the use of food color [52].

The mechanical and physical characteristics of the film might be impacted by the roughness of its surface. The values of R_a_ and Z values (as shown in Table 2) for the G+CS, G+CS+R, G+CS+G, G+CS+B, and G+CS+Y films were 10.77 nm/94.83 nm, 10.83 nm/291.41 nm, 31.23 nm/258.72 nm, 37.95 nm/443.92 nm, and 77.17 nm/471.30 nm, respectively. The R_q_ values for G+CS, G+CS+R, G+CS+G, G+CS+B, and G+CS+Y were 13.18 nm, 17.55 nm, 43.98 nm, 54.60 nm, and 93.38 nm, respectively. G+CS+Y and G+CS+B both had rougher surfaces than the rest. Gelatin–cornstarch films showed the least amount of surface roughness when red and green were introduced. They also exhibited a smooth surface, but any additional food color, such as blue and yellow, decreased the smoothness of the film surface [37,53]. 

### 3.7. Thermal Stability of Film

**Figure 9:**
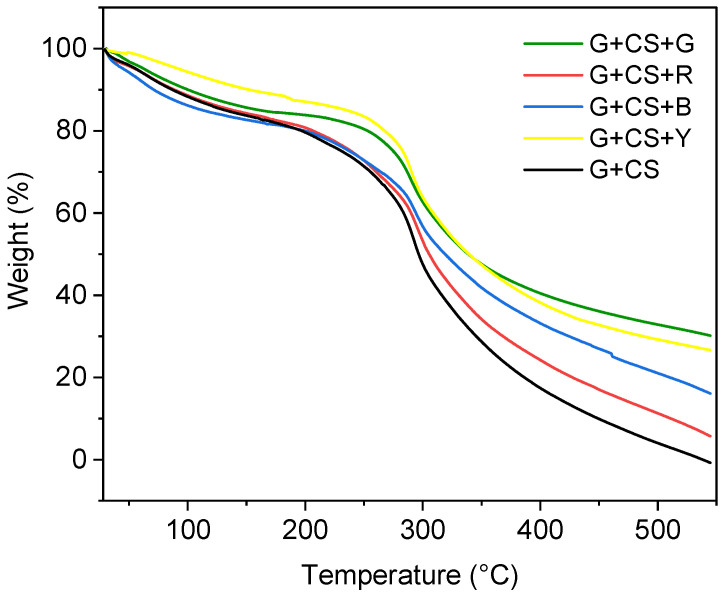
Thermogravimetric analysis (TGA) of the simple gelatin–cornstarch-based film (black line) and the films with food color addition. The blue curve represents the G+CS+B film, the red curve represents the G+CS+R film, the green curve represents the G+CS+G film, and the yellow curve represents the G+CS+Y film.

The thermal stability of the gelatin–cornstarch-based films was evaluated using TGA. According to three primary phenomena, all TGA curves (Figure 9) showed a similar mass loss behavior. Three stages of deterioration were tracked for each film. The first stage was captured between 50 °C and 100 °C (with each film decomposing by roughly 5%) [54]. The initial mass loss can be attributed to the polymeric structures’ evaporating water. The second stage, which was seen at temperatures between 120 and 200 °C, may be the breakdown of glycerol. The denaturation of the gelatin’s polymeric structure and food color was the primary source of the mass loss, which was seen between 240 and 300 °C. In the case of G+CS, almost all the mass of the sample was absent at high temperatures (>500 °C), which suggest that films without color are not as stable at temperature as films with color additions. All the organic material escapes leaving as CO_2_ [40,55]. This is also demonstrated in the results in Figure 9, which suggest that adding food colors to the gelatin matrix increases the thermal stability. In this stage, the pure gelatin–cornstarch extract film demonstrated a similar stability to the controlled gelatin–cornstarch-based film. These stages of deterioration matched those of the gelatin–cornstarch-based, film with color addition. The powder concentrations for G+CS, G+CS+R, G+CS+B, G+CS+Y, and G+CS+G were 0.6%, 6.1%, 18.9%, 26.5, and 30.51%, respectively, at the completion of thermal degradation. In general, the addition of food coloring had little effect on the thermal stabilities of films made using gelatin and cornstarch [56]. 

### 3.8. Barrier Effect on Film

**Figure 10:**
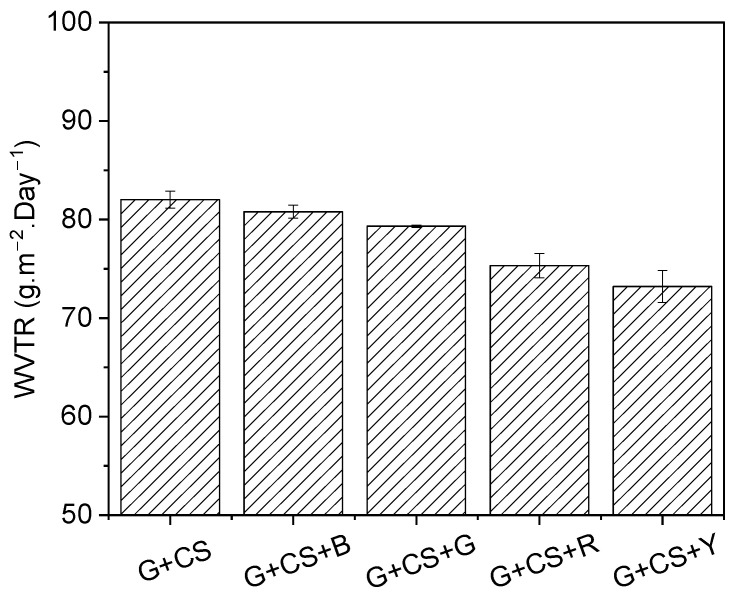
Effect of food color on the water vapor transmission rate (WVTR) of gelatin–cornstarch-based edible films.

Figure 10 shows the impact of food color on the gelatin–cornstarch-based edible film’s WVTR. The edible film’s capacity to store water vapor is indicated by its WVTR value. A smaller WVTR number indicates that water vapor will find it harder to pass through, extending the product’s shelf life. Figure 10 shows that the value of WVTR tended to decrease as food color was added to the gelatin-based film that was made of cornstarch and gelatin [57]. The samples with yellow food color had the lowest WVTR value (73.21 g·m^−2^·day^−1^). When food color concentrations of blue, green, red, and yellow were added, the intermolecular association was reduced and molecule mobility, which aids in water vapor migration, decreased. Additionally, glycerol possesses hydrophilic qualities that provide the film’s polar characteristics. An increased intermolecular distance results in fewer internal hydrogen bonds and intermolecular stresses on the matrix of edible films. Moisture could not flow through and enhance permeability when that distance is maintained. This outcome is in line with [58], who claim that open spaces in the film matrix can easily diffuse water vapor due to the lower molecular density brought about by the presence of hydrophilic groups in glycerol [59].

### 3.9. Real-Time Test

**Figure 11:**
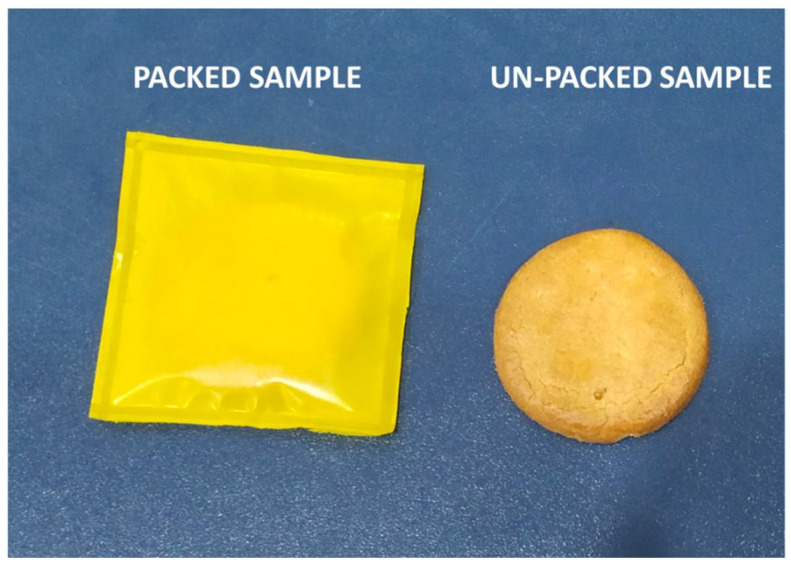
Pictorial representation of a biscuit packed in the G+CS+Y film and an unpacked biscuit sample.

**Figure 12:**
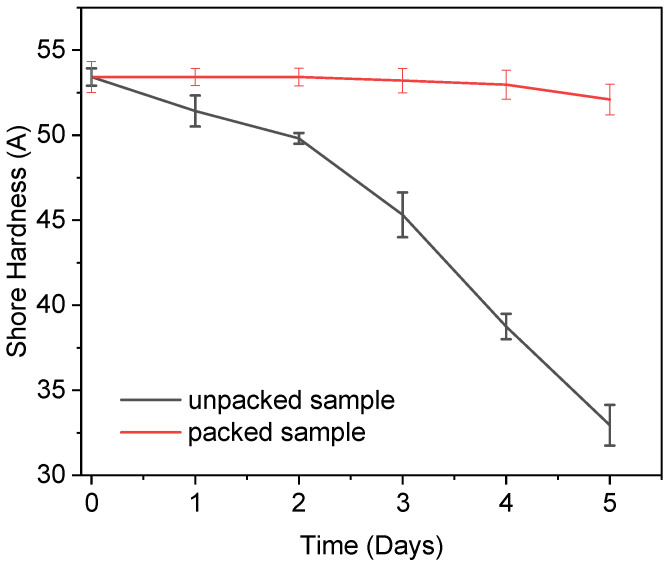
The graph represents the shore hardness vs. time graph in which the red line indicates the packed sample’s shore hardness A at 5 days and the black line indicates the unpacked sample’s shore hardness A after 5 days.

The shore hardness test was used to differentiate between samples that had been packed and ones that had not (see Figure 11). The results shore hardness testing of the packed and unpacked samples are shown in Figure 12. The graph shows that, although the sample that had been packed retained its hardness, the sample that had been unpacked continuously lost hardness owing to moisture penetration. The hardness test reveals that packed samples were superior to unpacked samples. The unpacked sample had a rating on the shore hardness scale A of below 30, which is regarded as being a soft material [60].

## 4. Conclusions

The major objective of this research was to create environmentally friendly and attractive edible films utilizing common household items such as gelatin and cornstarch, together with various food colors. The findings show that adding food color to gelatin–cornstarch-based films considerably enhanced their basic characteristics, such as resistance to moisture, mechanical characteristics, and thermal stability. According to the FT-IR data, the addition of food color to the cornstarch and gelatin-based film did not alter the film’s basic compositional state. The original gelatin–cornstarch-based film was more transparent but less bendable as compared to the shaded films. Even at low concentrations of food color, the presence of food color in the modified gelatin–cornstarch-based films provided a notable increase in the mechanical performance. With the addition of food color concentrations, the WVTR decreased by a factor of around one. The amount of food color added enhanced the roughness of the film’s surface and the films remained smooth and defect free. The shore hardness findings demonstrate that the colored films’ packaging capabilities were significantly improved. This method is likely to be used in the future to create eco-friendly edible films for the coating and packaging of foods.

## Figures and Tables

**Table 1:** Concentration of edible film with and without different food colors.

Sample	Composition	
G+CS	Gelatin (6 wt.%) + Cornstarch (2 wt.%) + Glycerin (3 wt.%)	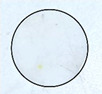
G+CS+Y	Gelatin (6 wt.%) + Cornstarch (2 wt.%) + Glycerin (3 wt.%) + yellow color liquid (1 wt.%)	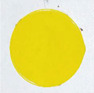
G+CS+R	Gelatin (6 wt.%) + Cornstarch (2 wt.%) + Glycerin (3 wt.%) + red color liquid (1 wt.%)	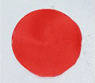
G+CS+G	Gelatin (6 wt.%) + Cornstarch (2 wt.%) + Glycerin (3 wt.%) + green color liquid (1 wt.%)	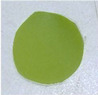
G+CS+B	Gelatin (6 wt.%) + Cornstarch (2 wt.%) + Glycerin (3 wt.%) + blue color liquid (1 wt.%)	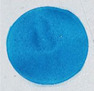

**Table 2:** Comparison of R_a_, R_q_, and Z values obtained from AFM images of different films.

Sample	R_a_ (nm)	R_q_ (nm)	R_z_ (nm)
G+CS	10.77	13.18	94.83
G+CS+R	10.83	17.55	291.41
G+CS+G	31.23	43.98	258.72
G+CS+B	37.95	54.60	443.92
G+CS+Y	77.17	93.38	471.30

## Data Availability

Data are available upon request from the corresponding authors.
